# Poly(dA:dT) Suppresses HSV-2 Infection of Human Cervical Epithelial Cells Through RIG-I Activation

**DOI:** 10.3389/fimmu.2020.598884

**Published:** 2021-02-04

**Authors:** Dan-Dan Shao, Feng-Zhen Meng, Yu Liu, Xi-Qiu Xu, Xu Wang, Wen-Hui Hu, Wei Hou, Wen-Zhe Ho

**Affiliations:** ^1^School of Basic Medical Sciences, Wuhan University, Wuhan, China; ^2^Department of Pathology and Laboratory Medicine, Lewis Katz School of Medicine, Temple University, Philadelphia, PA, United States

**Keywords:** herpes simplex virus type 2, human cervical epithelial cells, poly(dA:dT), interferon, interferon-stimulated gene, retinoic acid-inducible gene-I

## Abstract

Epithelial cells of the female reproductive tract (FRT) participate in the initial innate immunity against viral infections. Poly(dA:dT) is a synthetic analog of B form double-stranded (ds) DNA which can activate the interferon (IFN) signaling pathway-mediated antiviral immunity through DNA-dependent RNA Polymerase III. Here we investigated whether poly(dA:dT) could inhibit herpes simplex virus type 2 (HSV-2) infection of human cervical epithelial cells (End1/E6E7). We demonstrated that poly(dA:dT) treatment of End1/E6E7 cells could significantly inhibit HSV-2 infection. Mechanistically, poly(dA:dT) treatment of the cells induced the expression of the intracellular IFNs and the multiple antiviral IFN-stimulated genes (ISGs), including IFN-stimulated gene 15 (ISG15), IFN-stimulated gene 56 (ISG56), 2’-5’-oligoadenylate synthetase 1 (OAS1), 2’-5’-oligoadenylate synthetase 2 (OAS2), myxovirus resistance protein A (MxA), myxovirus resistance protein B (MxB), virus inhibitory protein, endoplasmic reticulum-associated, IFN-inducible (Viperin), and guanylate binding protein 5 (GBP5). Further investigation showed that the activation of RIG-I was largely responsible for poly(dA:dT)-mediated HSV-2 inhibition and IFN/ISGs induction in the cervical epithelial cells, as RIG-I knockout abolished the poly(dA:dT) actions. These observations demonstrate the importance for design and development of AT-rich dsDNA-based intervention strategies to control HSV-2 mucosal transmission in FRT.

## Introduction

Herpes simplex virus type 2 (HSV-2) is the leading cause of genital herpes and the most commonly sexual transmitted virus. It is estimated that 417 million people aged 15–49 (11%) worldwide are infected with HSV-2 infection in 2012 (1). Importantly, the epidemiological and biological studies have shown a strong association between human immunodeficiency virus 1 (HIV-1) and HSV-2 infection. HSV-2 infection increases the risk of HIV-1 acquisition by approximately three-fold (2) and facilitates transmissibility of HIV-1 up to five-fold through genital ulcers (3). In turn, HIV-1 infection enhances HSV-2 shedding frequency and quantity (4, 5), people with HIV-1-related immunosuppression can have severe HSV-2 diseases (6, 7).

Epithelial cells in the female reproductive tract (FRT) are the first barrier to pathogen invasion. At cellular level, epithelial cells constitute a unique microenvironment and participate in FRT innate immunity against viral infections, including HSV-2 (8, 9). HSV-2 primarily infects genital epithelium and replicates within the vaginal keratinocytes (10). Human cervical epithelial cells have been extensively used to study FRT-mediated immunity against viral infections (11–14). Studies have shown that these cells could be immunologically activated and produced the multiple antiviral factors against HSV-2 (12) and HIV-1 (15). As the outmost layer cells in FRT, human cervical epithelial cells are the first to contact with invading microbes. Thus, understanding the processes and mechanisms of these cells-mediated innate immunity against viral infections is of importance and significance.

DNA-dependent RNA polymerase III (RNA Pol III) is involved in DNA-mediated innate immunity response by converting AT-rich DNA into a RNA intermediate which can be recognized by RIG-I, resulting in the activation of interferon (IFN) signaling pathway. Poly(dA:dT) is a synthetic analog of B form double stranded DNA (dsDNA) can be sensed by the RNA Pol III and then recognized by cytosolic RNA sensor RIG-I, eliciting an intracellular immune response to control virus replication (16–18). IFN regulatory factor 3 (IRF3) and IRF7 are the crucial transcription factors involved in RIG-I signaling pathway (19). It has been reported that the association of RIG-I with pre-genomic RNA can induce type III IFN and inhibit hepatitis B virus (20). Type III IFN, also known as IFN lambda (IFN-λ), can induce expression of ISGs and exert antiviral properties similar to type I IFNs (21).

Studies have shown that activation of IFN-dependent innate immune defense through RIG-I signaling pathway is vital in antiviral response of epithelial cells (22–24). However, we know little about whether poly(dA:dT) has the ability to activate the intracellular antiviral immunity of human cervical epithelial cells, an essential component of the mucosal defense mechanisms in the FRT. In this study, we examined whether poly(dA:dT) has the ability to induce the intracellular antiviral factors against HSV-2 infection of human cervical epithelial cells. We also explored the cellular and molecular mechanisms underlying poly(dA:dT)-mediated IFN/ISG induction and HSV-2 inhibition in these cells.

## Materials and Methods

### Cell Lines and Virus

End1/E6E7 cell line was established from normal human endocervical epithelia immortalized by expression of human papillomavirus 16/E6E7. End1/E6E7 cells have exactly the same cytokeratin and involucrin patterns as primary End1 cells and the morphological and immunocytochemical characteristics of the End1/E6E7 cells closely resembled those of origin and primary cultures. Therefore, End1/E6E7 cells provide the basis for valid reproducible *in vitro* models for studies on cervicovaginal physiology and infections and for testing pharmacological agents for intravaginal application (25). In addition, End1/E6E7 cell line has been broadly used as an *in vitro* model of human female reproductive tract (13, 26, 27). End1/E6E7 cells were cultured in keratinocyte growth medium (Gibco, USA) supplemented with the provided recombinant epidermal growth factor (0.1ng/ml) and bovine pituitary extract (50µg/ml). African green monkey kidney epithelial cells (Vero) and 293T cells were cultured in Dulbecco’s modified Eagle’s culture medium (DMEM, Gibco) supplemented with 10% fetal bovine serum (FBS, Gibco) at 37°C in a humidified atmosphere of 5% CO_2_. HSV-2 G strain was provided by Dr. Qinxue Hu (State Key Laboratory of Virology, Wuhan Institute of Virology, Chinese Academy of Sciences, China). The HSV-2 G strain was propagated at a low multiplicity of infection (MOI = 0.001) in Vero cells.

### Plasmids and Reagents

LentiCRISPRv2-puro, psPAX2, and pMD2.G plasmids were provided by Dr. Jian Huang (Department of Pathology and Laboratory Medicine, Temple University, USA). PE Annexin V Apoptosis Detection Kit I was purchased from BD (Pharmingen, USA). Poly(dA:dT), LyoVec, and puromycin were purchased from InvivoGen (San Diego, CA, USA). Lipofectamine 3000 Reagent was purchased from Thermo Fisher Scientific (Carlsbad, CA, USA). Antibodies against RIG-I, DNA sensors, ISGs, signal transducers and activators of transcription (STATs), and IRFs were purchased from Cell Signaling Technology (Danvers, MA, USA). Antibodies against HSV-1+HSV-2 gD, HSV-1+HSV-2 gB were purchased from Abcam (Cambridge, UK). Antibody to GAPDH was purchased from Proteintech (Chicago, USA).

### Cell Viability Assay

The cytotoxic effect of poly(dA:dT) was evaluated by the MTT assay based on the manufacturer’s instruction. End1/E6E7 cells were seeded in 96-well plate (1×10^4^ cells/well) treated with different concentrations of poly(dA:dT) for 72h. Cells were then incubated with MTT working solution (0.5mg/ml) for 4h at 37°C in darkness. The formation of soluble formazan from MTT was measured by spectrophotometric determination of absorption at 490nm using a 96-well plate reader (SpectraMax i3, Molecular Devices, Sunnyvale, CA, USA).

### Flow Cytometry Analysis of Apoptosis

We used Annexin V/7-AAD assay to measure the apoptosis effect of poly(dA:dT) on End1/E6E7 cells. Cells were seeded in 24-well plate (2×10^5^ cells/well) and treated with different concentrations of poly(dA:dT) for 72h. Cells were washed twice with cold phosphate-buffered saline (PBS) and then resuspended in 1×binding buffer at a concentration of 1×10^5^ cells/ml. Annexin V-PE (2.5µl) and 7-AAD (5µl) were added and then incubated for 15min at room temperature without light, finally analyzed by FCM (FACScan, Becton Dickinson, San Jose, CA).

### RNA Extraction and Real-Time PCR

Total cellular RNAs from the cells were extracted using TRI-Reagent^®^ (Molecular Research Center, Cincinnati, OH) according to the manufacturer’s instruction. Total RNAs were subjected to reverse transcription reaction using the random primer, dNTPs, M-MLV reverse transcriptase and RNase inhibitor (Promega Co., Madison, WI) to generate complementary DNA (cDNA). cDNA was then used as a template for real-time PCR which was performed with IQ SYBR Green supermix (Bio-Rad Laboratories, Hercules, CA). The level of GAPDH mRNA was used as an endogenous reference to normalize the quantities of target mRNAs. The sequences of oligonucleotide primers are shown in **Table 1**.

**Table 1:** Primer sets for real-time PCR.

Primer	Accession No.	Orientation	Sequences	Product (bp)
GAPDH	NM_002046	Sense Antisense	5’-GGTGGTCTCCTCTGACTTCAACA-3’ 5’-GTTGCTGTAGCCAAATTCGTTGT-3’	127
HSV-2 ICP0	D10471.1	Sense Antisense	5’-GTGCATGAAGACCTGGATTCC-3’ 5’-GGTCACGCCCACTATCAGGTA-3’	82
HSV-2 ICP27	D10471.1	Sense Antisense	5’-TTCTGCGATCCATATCCGAGC-3’ 5’-AAACGGCATCCCGCCAAA-3’	101
HSV-2 ICP8	D10658.1	Sense Antisense	5’-AGGACATAGAGACCATCGCGTTCA-3’ 5’-TGGCCAGTTCGCTCACGTTATT-3’	99
HSV-2 gC	AJ297389.1	Sense Antisense	5’-AAATCCGATGCCGGTTTCCCAA-3’ 5’-TTACCATCACCTCCTCTAAGCTAGGC-3’	120
HSV-2 gD	K02373.1	Sense Antisense	5’-ATCCGAACGCAGCCCCGC-3’ 5’-TCTCCGTCCAGTCGTTTAT-3’	142
HSV-2 DNA polymerase	M16321.1	Sense Antisense	5’-GCTCGAGTGCGAAAAAACGTTC-3’ 5’-CGGGGCGCTCGGCTAAC-3’	215
IFN-β	NM_002176.4	Sense Antisense	5’-GCCGCATTGACCATCTATGAGA-3’5’-GAGATCTTCAGTTTCGGAGGTAAC-3’	346
IFN-λ1	NM_172140	Sense Antisense	5’-CTTCCAAGCCCACCCCAACT-3’ 5’-GGCCTCCAGGACCTTCAGC-3’	142
IFN-λ2/3	NM_172139	Sense Antisense	5’-TTTAAGAGGGCCAAAGATGC-3’ 5’-TGGGGCTGAGGCTGGATACAG-3’	267
IRF3	NM_001571.1	Sense Antisense	5’-ACCAGCCGTGGACCAAGAG-3’ 5’-TACCAAGGCCCTGAGGCAC-3’	65
IRF7	NM_001572	Sense Antisense	5’-TGGTCCTGGTGAAGCTGGAA-3’ 5’-GATGTCGTCATAGAGGCTGTTGG-3’	134
STAT1	NM_007315	Sense Antisense	5’-CCGTGGCACTGCATACAATC-3’ 5’-ACCATGCCGAATTCCCAAAG-3’	187
STAT2	XM_017019904.1	Sense Antisense	5’-CCCCATCGACCCCTCATC-3’ 5’-GAGTCTCACCAGCAGCCTTGT-3’	69
STAT3	NM_003150	Sense Antisense	5’-CTGCCCCATACCTGAAGACC-3’ 5’-TCCTCACATGGGGGAGGTAG-3’	162
ISG15	NM_005101	Sense Antisense	5’-GGCTGGGACCTGACGGTGAAG-3’ 5’-GCTCCGCCCGCCAGGCTCTGT-3’	492
ISG56	NM_001270930	Sense Antisense	5’-TTCGGAGAAAGGCATTAGA-3’ 5’-TCCAGGGCTTCATTCATAT-3’	85
OAS1	NM_001032409	Sense Antisense	5’-AGAAGGCAGCTCACGAAACC-3’ 5’-CCACCACCCAAGTTTCCTGTA-3’	71
OAS2	XM_011538415.1	Sense Antisense	5’-CAGTCCTGGTGAGTTTGCAGT-3’ 5’-ACAGCGAGGGTAAATCCTTGA-3’	146
MxA	XM_005260982.2	Sense Antisense	5’-GCCGGCTGTGGATATGCTA-3’ 5’-TTTATCGAAACATCTGTGAAAGCAA-3’	69
MxB	XM_005260983.4	Sense Antisense	5’-CAGCAGACGATCAACTTGGTG-3’ 5’-CATGACGCTTTTCTCAGTGCC-3’	159
Viperin	NM_001318443.1	Sense Antisense	5’-TGGGTGCTTACACCTGCTG-3’ 5’-TGAAGTGATAGTTGACGCTGGT-3’	235
GBP5	NM_001134486.2	Sense Antisense	5’-TGGCAAATCCTACCTGATGA-3’ 5’-CCATATCCAAATTCCCTTGG-3’	97
RIG-I	NM_014314.4	Sense Antisense	5’-CTTGGCATGTTACACAGCTGAC-3’ 5’-GCTTGGGATGTGGTCTACTCA-3’	104
IFI16	NM_001376589.1	Sense Antisense	5’-CCGTTCATGACCAGCATAGG-3’ 5’-TCAGTCTTGGTTTCAACGTGGT-3’	106
cGAS	NP_612450.2	Sense Antisense	5’-GGGAGCCCTGCTGTAACACTTCTTAT-3’ 5’-CCTTTGCATGCTTGGGTACAAGGT-3’	186
STING	NP_938023.1	Sense Antisense	5’-ACTGTGGGGTGCCTGATAAC-3’ 5’-TGGCAAACAAAGTCTGCAAG-3’	197
DAI	NM_001160418.2	Sense Antisense	5’-CAACAACGGGAGGAAGACAT-3’ 5’-TCATCTCATTGCTGTGTCCC-3’	499
AIM2	NM_004833.3	Sense Antisense	5’-TAGCGCCTCACGTGTGTTAG-3’ 5’-TTGAAGCGTGTTGATCTTCG-3’	103
DHX29	NM_001345965.2	Sense Antisense	5’-TCAGCACCTGGGAGCTACTT-3’ 5’-TCTGCATCACTCCACTCCAG-3’	111

### *In Vitro* Antiviral Assay

End1/E6E7 cells were pretreated with poly(dA:dT) for 24h and infected with HSV-2 (MOI = 0.001) for 2h. The cells were then washed to remove unattached viruses and subsequently cultured for 48h. HSV-2 genome DNAs from HSV-2-infected cells and culture supernatant were extracted with DNA lysis buffer as previously described (12) and subjected to the real-time PCR. HSV-2 gD standards with known copy numbers were used to quantify HSV-2 gD copies in the culture supernatant. Total proteins were extracted from End1/E6E7 cells and subjected to the Western blot. In addition, the antiviral effect of poly(dA:dT) under different treatment conditions (before, simultaneously and after HSV-2 infection) was evaluated. Briefly, End1/E6E7 cells were pretreated with poly(dA:dT) (0.5µg/ml) for 24h, then infected with HSV-2 (before); End1/E6E7 cells were simultaneously (simul) treated with poly(dA:dT) and infected with HSV-2; End1/E6E7 cells were first infected with HSV-2 for 2h, then washed and treated with poly(dA:dT) (after). At 48h post infection, both HSV-2 genomic DNA and total proteins were extracted from End1/E6E7 cells and subjected to the real-time PCR or Western blot assay.

### CRISPR Cas9

We used the CRISPR Cas9 system to abrogate RIG-I expression in End1/E6E7 cells. Briefly, we designed gRNA (5’-GGGTCTTCCGGGATATAATCC-3’) targeting the conserved sites in human RIG-I genomic sequences based on CCTop (https://crispr.cos.uni-heidelberg.de/). The gRNA was then subcloned into the lentiCRISPRv2 plasmid to obtain the lentiCRISPRv2-gRNA clone that expressed both Cas9 and gRNA according to the publications’ instruction (28, 29). Then lentiCRISPRv2-gRNA plasmid and two packaging plasmids, psPAX2 and pMD2.G, were co-transfected into 293T cells to obtain lentivirus. LentiCRISPRv2 was used as an empty vector control. Three days after the lentivirus infection, End1/E6E7 cells were cultured with puromycin (0.5µg/ml) containing medium for 14 days. Total cellular proteins were then collected and subjected to Western blot for RIG-I protein expression.

### Western Blot

Total cell lysates were prepared with the cell extraction buffer (Invitrogen, Shanghai, China) with 1% protease inhibitor cocktail (Sigma, MO) and 1% phosphatase inhibitor mixture (Applygen, Beijing, China). The total proteins were quantified by a BCA protein assay kit (Beyotime Institute of Biotechnology, Shanghai, China), and equal amount of proteins were separated on SDS-PAGE. After being transferred to a PVDF membrane (Millipore, Germany), non-specific sites were blocked with 5% non-fat milk for 3h prior to incubating with primary antibodies at 4°C overnight. The membranes were washed with TBST and further incubated with horseradish peroxidase-conjugated second antibody. Blots were developed with SuperSignal West Pico Chemiluminescent Substrate (Thermo Fisher Scientific, Waltham).

### ELISA

IFN-β, IFN-λ1/3, and IFN-λ2 protein levels in cells culture supernatant were measured by ELISA kits (R&D system Inc., MH, USA). The assays were carried out according to the manufacturer’s instruction.

### Statistical Analysis

Data were presented as mean ± SD from at least three independent experiments, and statistical significance was analyzed by Student’s t-test using GraphPad Prism for Windows version 5.0 (GraphPad Software Inc., San Diego, CA). Statistical significance was defined as *P* < 0.05 or *P* < 0.01.

## Results

### Poly(dA:dT) Has Little Cytotoxicity Effect on End1/E6E7 Cells

**Figure 1:**
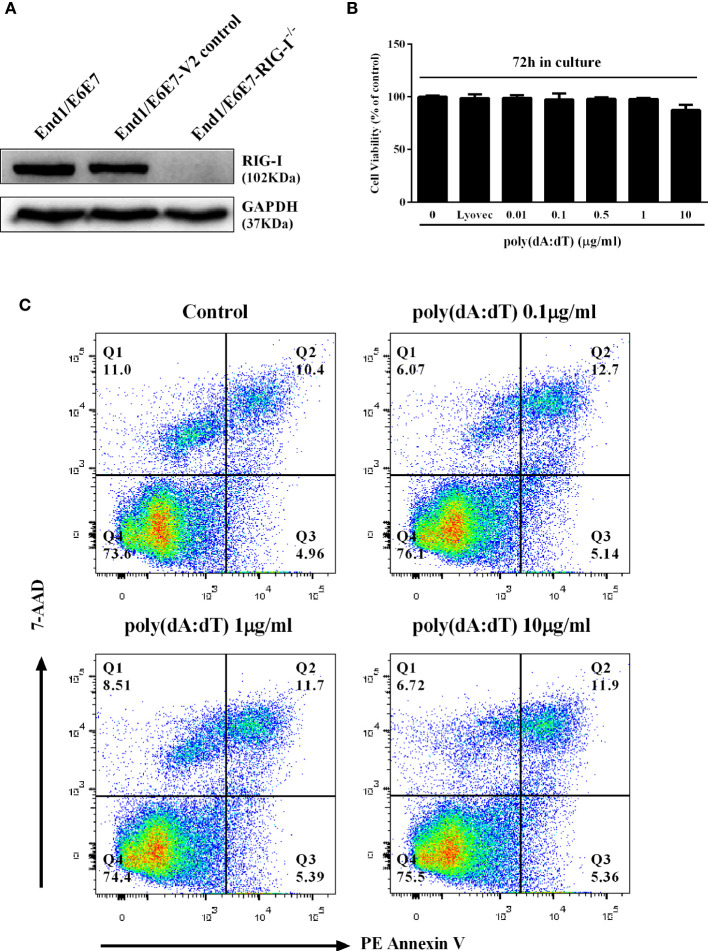
Effect of poly(dA:dT) on End1/E6E7 cells. **(A)** Cell lysates of End1/E6E7 cells, End1/E6E7 V2 control cells and RIG-I knockout End1/E6E7 cells were subjected to Western blot assay for RIG-I expression and GAPDH was used as a protein loading control. **(B, C)** End1/E6E7 cells were treated with poly(dA:dT) at the indicated concentrations, and cell viability was assessed by MTT assay **(B)** and annexin V/7-AAD assay **(C)** 72h post poly(dA:dT) treatment.

We first examined the effect of poly(dA:dT) on the viability and apoptosis of End1/E6E7 cells. As shown in **Figures 1B, C**, little cytotoxic and apoptosis effect was observed in End1/E6E7 cells treated with poly(dA:dT) at the dose as high as 10µg/ml.

### Poly(dA:dT) Inhibits HSV-2 Infection of End1/E6E7 Cells

**Figure 2:**
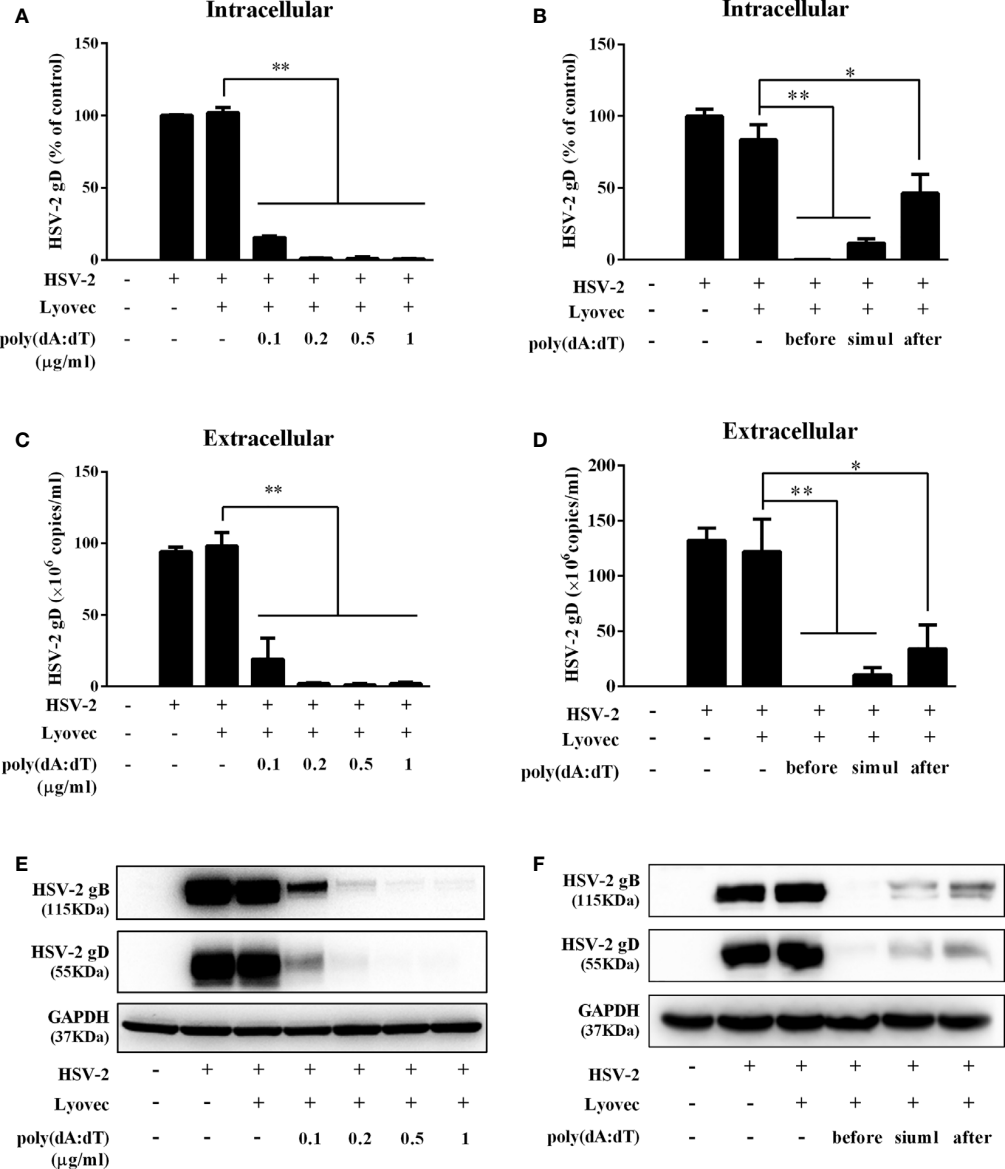
Poly(dA:dT) inhibits HSV-2 infection. **(A, C, E)** End1/E6E7 cells were pretreated with poly(dA:dT) at indicated concentrations for 24h prior to HSV-2 (MOI = 0.001) infection. Forty-eight hours after HSV-2 infection, **(A)** intracellular DNA, **(C)** extracellular DNA, and **(E)** total cellular proteins were collected and subjected to the real-time PCR or Western blot for HSV-2 gene expression. **(B, D, F)** End1/E6E7 cells were treated with either poly(dA:dT) (0.5μg/ml) for 24h prior to HSV-2 (MOI=0.001) infection (before) or poly(dA:dT) and infected with HSV-2 simultaneously (simul) or infected with HSV-2 for 2h prior to poly(dA:dT) treatment (after). At 48h post HSV-2 infection, **(B)** intracellular DNA, **(D)** extracellular DNA, and **(F)** total cellular proteins were collected and analyzed by the real-time PCR or Western blot for HSV-2 gene expression. Data shown are the mean ± SD of three independent experiments. Asterisks indicate statistically significant differences. (*P < 0.05, **P < 0.01).

**Figure 3:**
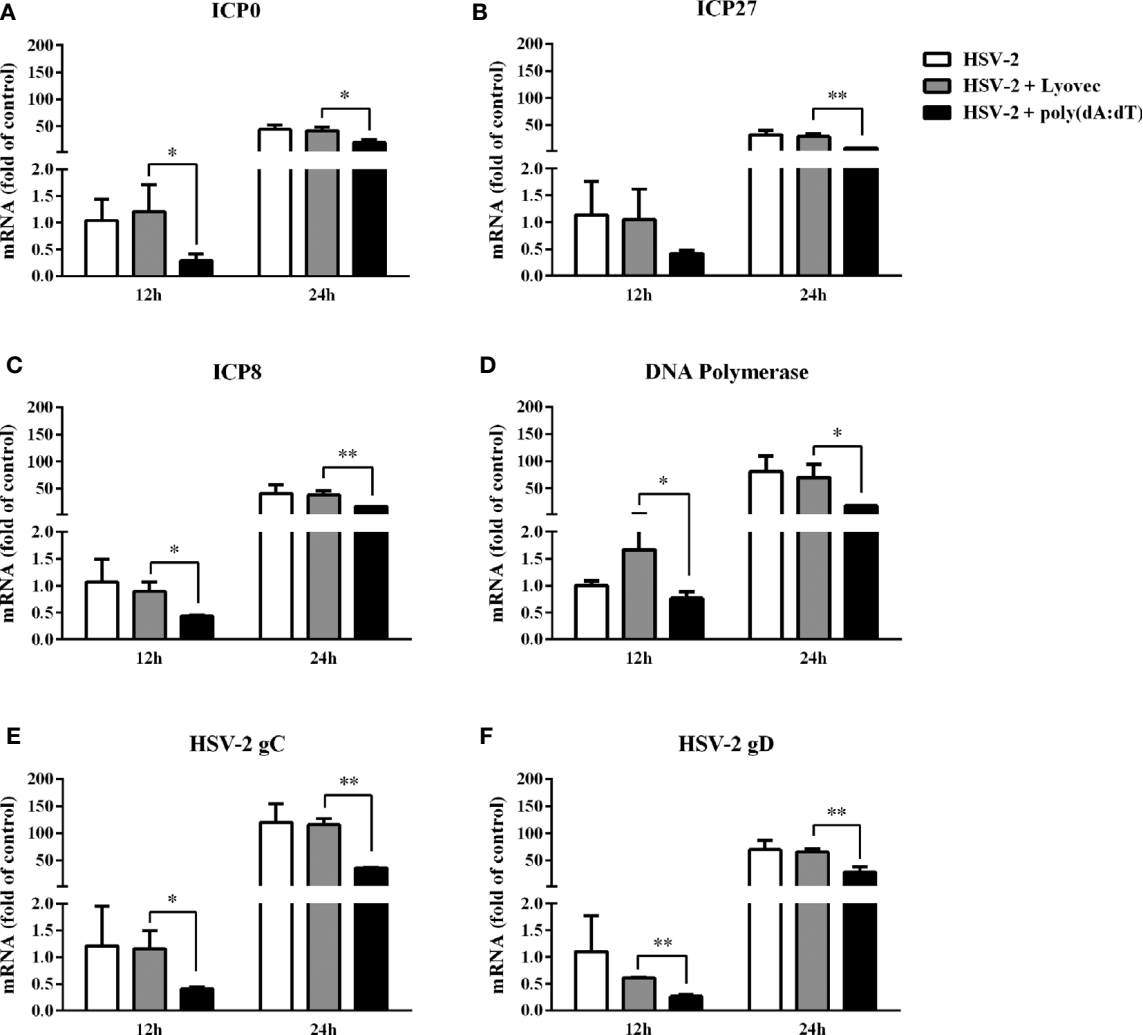
Effect of poly(dA:dT) on HSV-2 gene expression. End1/E6E7 cells were transfected with or without poly(dA:dT) (0.5μg/ml) at indicated concentrations for 24h prior to HSV-2 infection (MOI = 0.001). Cellular RNAs were collected from the virus-infected cells at 12 or 24h post infection and subjected to the real-time PCR for HSV-2 immediate early genes **(A, B)**, early genes **(C, D)** and late genes **(E, F)** expression. The results were measured as HSV-2 gene levels relative (%) to control (without treatment, which is defined as 100%). Data are shown as mean ± SD of three independent experiments. Asterisks indicate statistically significant differences (*P < 0.05, **P < 0.01).

To determine the anti-HSV-2 effect of poly(dA:dT), End1/E6E7 cells were pretreated with poly(dA:dT) for 24h prior to HSV-2 infection. As shown in **Figures 2A, C, E**, cells transfected with poly(dA:dT) had lower levels of intracellular and extracellular HSV-2 DNA/protein than the control cells. This poly(dA:dT)-mediated HSV-2 inhibition was dose-dependent. To further determine the anti-HSV-2 effect of poly(dA:dT), End1/E6E7 cells were treated with poly(dA:dT) under different treatment conditions (before, simultaneously and after HSV-2 infection). As shown in **Figures 2B, D, F**, under all the three treatment conditions, poly(dA:dT) were able to significantly suppress HSV-2 infection at both DNA and protein levels. Pretreatment of the cells with poly(dA:dT) was the most effective in HSV-2 inhibition (**Figures 2B, D, F**). In addition, we examined the effect of poly(dA:dT) on several key HSV-2 genes, including two immediate early genes (IE: ICP0 and ICP27), two early genes (E: ICP8 and DNA polymerase) and two late genes (L: HSV-2 gC and gD). As shown in **Figure 3**, poly(dA:dT) could significantly inhibit the expression of HSV-2 IE (**Figures 3A, B**), E (**Figures 3C, D**) and L (**Figures 3E, F**) genes in the infected cells.

### Poly(dA:dT) Activates the JAK/STAT Signaling Pathway

**Figure 4:**
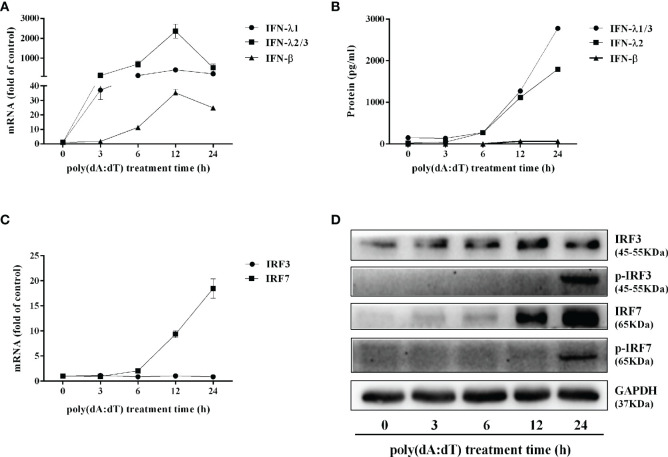
Effect of poly(dA:dT) on IFNs and IRFs expression. End1/E6E7 cells were transfected with or without poly(dA:dT) (0.5μg/ml) for the indicated times. **(A, C)** Total cellular RNAs were extracted and subjected to the real-time PCR for IFN-β, IFN-λ1, IFN-λ2/3, and IRF3, IRF7 expression. **(B)** The cell-free supernatant was subjected to ELISA assay to determine IFN-β, IFN-λ1/3, and IFN-λ2 protein levels. **(D)** Total cellular proteins were collected and subjected to Western blot with the antibodies against IRF3, IRF7, p-IRF3, p-IRF7, and GAPDH. Data are shown as mean ± SD of three independent experiments.

To determine the mechanisms by which poly(dA:dT) inhibits HSV-2 infection of End1/E6E7 cells (**Figures 2** and **3**), we examined whether poly(dA:dT) could activate IFN-based immunity in End1/E6E7 cells. As shown in **Figures 4A, B**, poly(dA:dT) induced the expression of IFN-β and IFN-λ at both mRNA (**Figure 4A**) and protein (**Figure 4B**) levels. These effects of poly(dA:dT) on IFN-β and IFN-λ induction were time-dependent. We next studied whether IRF3 and IRF7, the key positive regulators of the IFN signaling pathway, were involved in the IFN-β and IFN-λ induction by poly(dA:dT) in End1/E6E7 cells. As shown in **Figure 4D**, poly(dA:dT) facilitated the phosphorylation of both IRF3 and IRF7 (p-IRF3 and p-IRF7), which were positively associated with the treatment time of poly(dA:dT) in End1/E6E7 cells.

**Figure 5:**
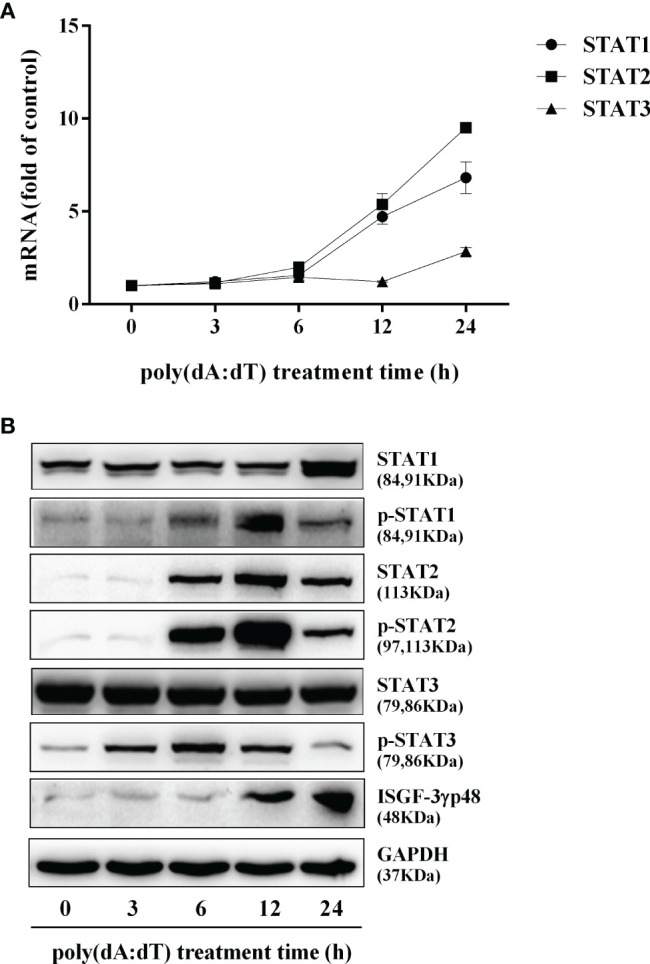
Effect of poly(dA:dT) on JAK/STAT signaling pathway. End1/E6E7 cells were transfected with or without poly(dA:dT) (0.5μg/ml) for the indicated times. **(A)** Total cellular RNAs were extracted and subjected to the real-time PCR for STAT1, STAT2, and STAT3 expression. **(B)** Total cellular proteins were collected and subjected to Western blot with the antibodies against STAT1, STAT2, STAT3, p-STAT1, p-STAT2, p-STAT3, ISGF-3γp48, and GAPDH. Data are shown as mean ± SD of three independent experiments.

**Figure 6:**
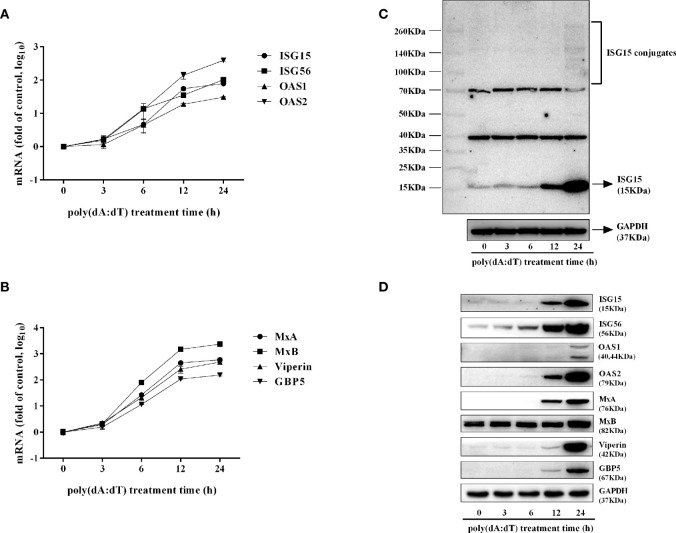
Effect of poly(dA:dT) on ISGs expression. End1/E6E7 cells were transfected with or without poly(dA:dT) (0.5μg/ml) for the indicated times. **(A, B)** Total cellular RNAs were isolated and subjected to the real-time PCR for ISG15, ISG56, OAS1, OAS2, MxA, MxB, viperin, and GBP5 expression. **(C, D)** Total cellular proteins were collected and subjected to Western blot with antibodies against ISG15 and ISG15-conjugates, ISG56, OAS1, OAS2, MxA, MxB, Viperin, GBP5, and GAPDH. Data shown represent the mean ± SD of three independent experiments.

To determine whether the induction of IFN-β and IFN-λ is responsible for the activation of JAK/STAT signaling pathway, we analyzed the impact of poly(dA:dT) on the expression of signal transducers and activators of transcription (STATs). As shown in **Figure 5**, poly(dA:dT) induced the mRNA expression of STAT1, STAT2, STAT3 (**Figure 5A**), and protein expression of p-STAT1, p-STAT2, p-STAT3, IFN-regulated transcription factor 3 (ISGF-3)-γp48 in a time-dependent fashion (**Figure 5B**). In addition, poly(dA:dT) treatment of End1/E6E7 cells also induced the expression of ISG15, ISG56, OAS1, OAS2, MxA, MxB, Viperin, and GBP5 at both mRNA (**Figures 6A, B**) and protein levels (**Figures 6C, D**).

### Poly(dA:dT) Induces RIG-I

**Figure 7:**
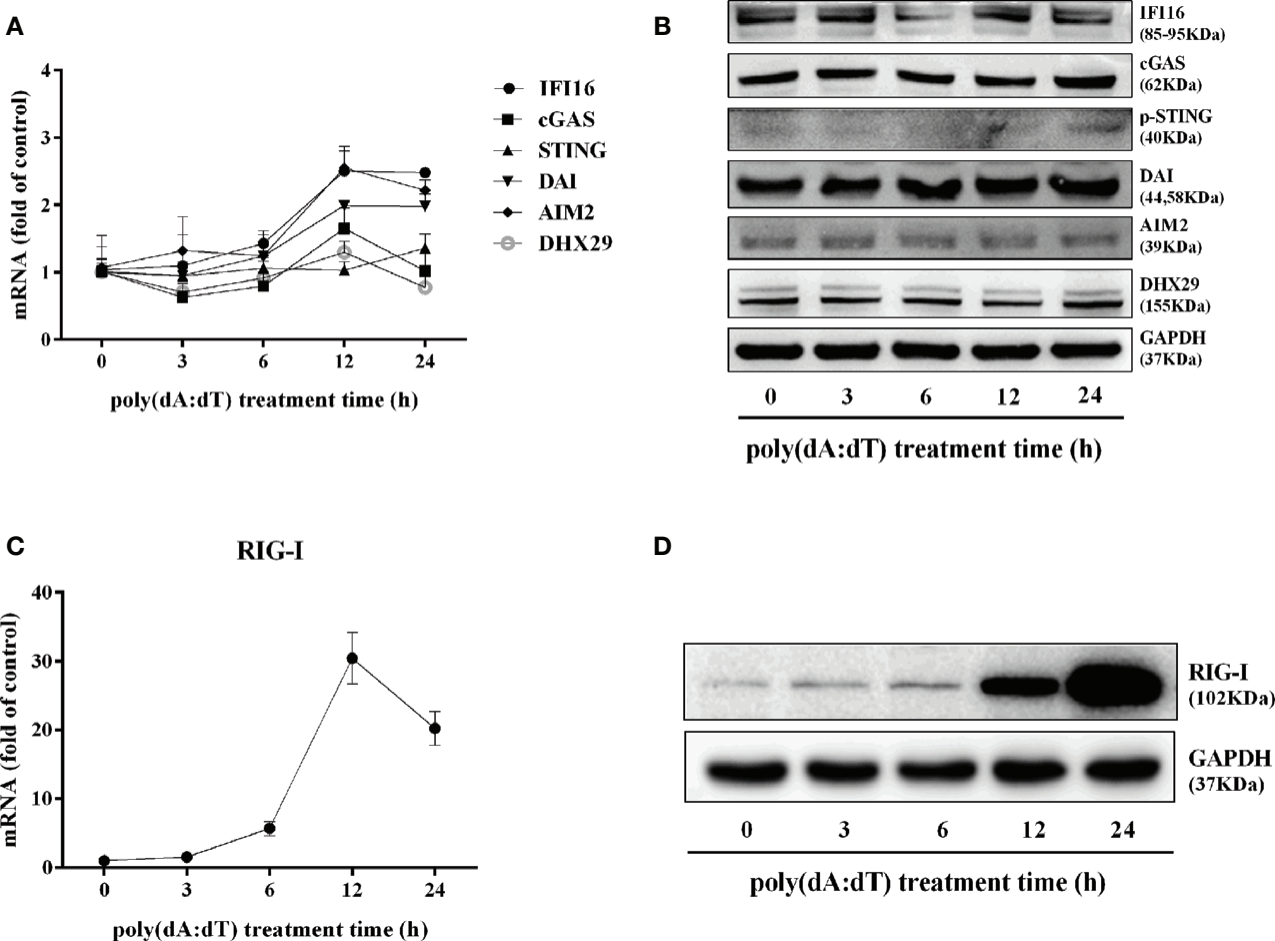
Effect of poly(dA:dT) on the DNA sensors and RIG-I. End1/E6E7 cells were transfected with or without poly(dA:dT) (0.5μg/ml) for the indicated times. **(A)** Total cellular RNAs were extracted and the messenger RNA (mRNA) levels of DNA sensors (IFI16, cGAS, STING, DAI, AIM2, and DHX29) were measured by the real-time PCR. **(B)** Total cellular proteins were collected and subjected to Western blot with the antibodies against IFI16, cGAS, p-STING, DAI, AIM2, DHX29, and GAPDH. **(C)** Total cellular RNAs were extracted and subjected to the real-time PCR for measuring RIG-I mRNA. **(D)** Total cellular proteins were collected and subjected to Western blot with the antibodies against RIG-I and GAPDH. Representative data are the mean ± SD of three independent experiments.

In addition to the DNA sensors (30–32), RIG-I (16, 17) is involved in sensing cytosolic DNA. We thus examined the effect of poly(dA:dT) on RIG-I expression in End/E6E7 cells. As shown in **Figures 7C, D**, poly(dA:dT) time-dependently enhanced the expression of RIG-I in the cells. In contrast, poly(dA:dT) had little effect on the expression of the key DNA sensors (IFI16, cGAS, phosphor-stimulator of IFN genes (p-STING), DAI, AIM2, and DExH-box helicase 29 (DHX29) (**Figures 7A, B**).

### RIG-I Knockout Significantly Compromises Poly(dA:dT)-Mediated HSV-2 Inhibition

**Figure 8:**
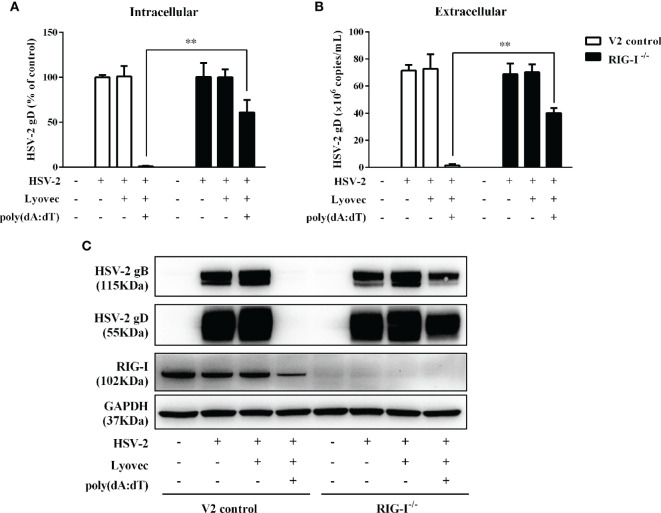
Effect of RIG-I knockout on poly(dA:dT)-mediated HSV-2 inhibition. End1/E6E7 control cells (V2 control) and RIG-I knockout End1/E6E7 cells (RIG-I^−/−^) were transfected with or without poly(dA:dT) (0.5μg/ml) for 24h prior to HSV-2 infection (MOI = 0.001). **(A, B)** Total DNAs extracted from cells (intracellular) and culture supernatant (Extracellular) were measured by the real-time PCR for HSV-2 gD expression. **(C)** Total cellular proteins were collected and subjected to Western blot with antibodies against HSV-2 gB, HSV-2 gD, RIG-I, and GAPDH. The results are the mean ± SD of three independent experiments. Asterisks indicate that the differences between the indicated groups are statistically significant (**P < 0.01).

**Figure 9:**
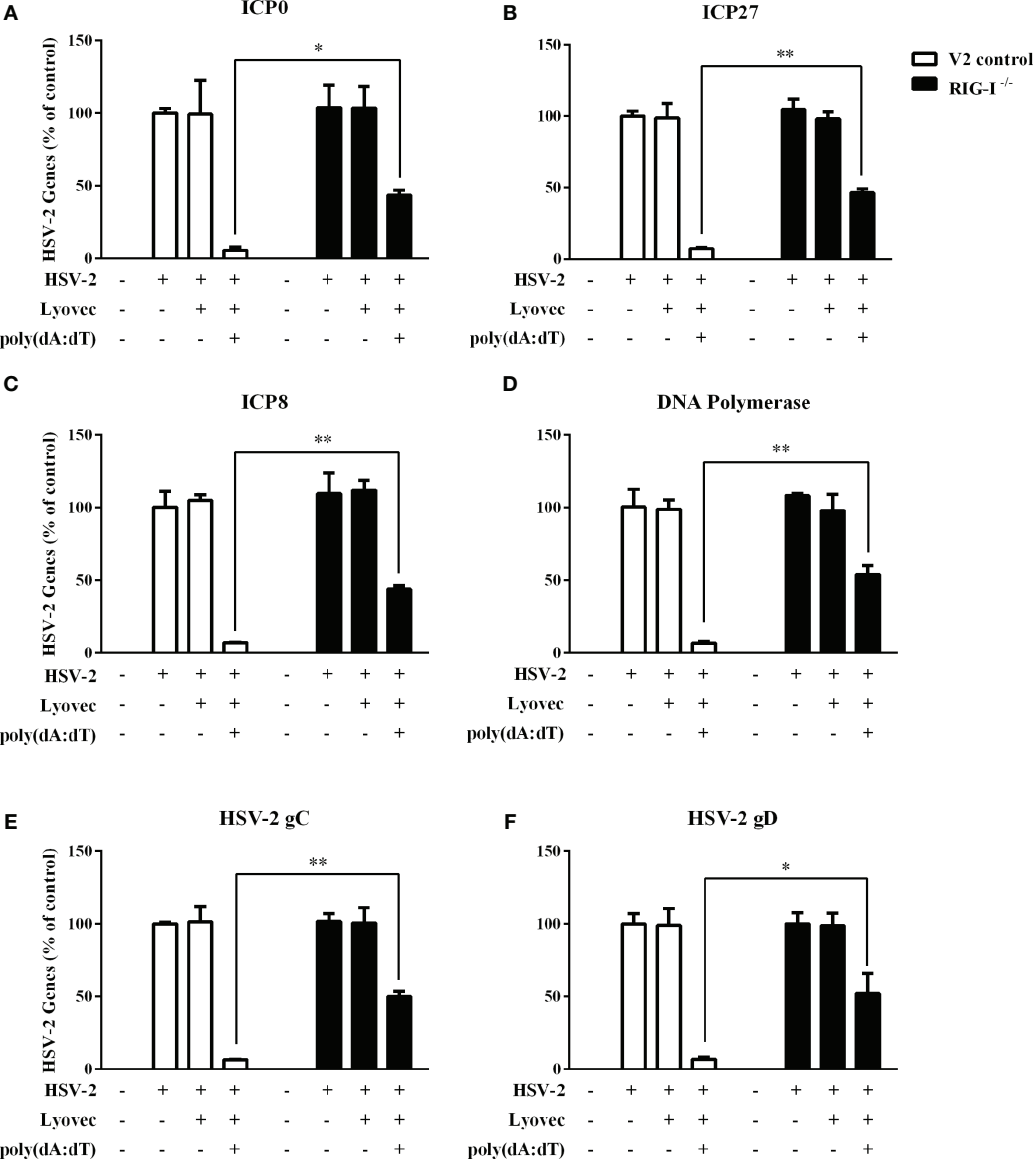
Effect of RIG-I knockout on poly(dA:dT)-mediated inhibtion of HSV-2 gene expression. End1/E6E7 control cells (V2 control) and RIG-I knockout End1/E6E7 cells (RIG-I^−/−^) were transfected with or without poly(dA:dT) (0.5μg/ml) for 24h prior to HSV-2 infection (MOI = 0.001). At 24h post HSV-2 infection, cellular RNAs were collected and subjected to the real-time PCR for HSV-2 immediate early genes **(A, B)**, early genes **(C, D)**, and late genes **(E, F)** expression. The results were measured as HSV-2 gene levels relative (%) to control (without treatment, which is defined as 100%). Data are shown as mean ± SD for three independent experiments. Asterisks indicate that the differences between the indicated groups are statistically significant (*P < 0.05, **P < 0.01).

To determine whether RIG-I plays a role in poly(dA:dT)-mediated HSV-2 inhibition, we constructed RIG-I knockout End1/E6E7 cells (RIG-I^−/−^) and End1/E6E7 V2 control cells (V2 control) through CRISPR Cas9 system (**Figure 1A**). As shown in **Figures 8A–C**, the levels of both HSV-2 DNA (**Figures 8A, B**) and protein (**Figure 8C**) in RIG-I^−/−^ cells were significantly higher than those in V2 control cells. In addition, we examined the impact of RIG-I knockout on poly(dA:dT)-mediated HSV-2 gene inhibition and observed that RIG-I^−/−^ cells has higher levels of HSV-2 IE, E and L genes expression than V2 control cells (**Figure 9**). The expression level of RIG-I in HSV-2 infected cells was lower than those in uninfected cells. Especially, the expression level of RIG-I in HSV-2 infected cells which were pretreated with poly(dA:dT) was significantly lower than those in uninfected cells.

### RIG-I Knockout Diminishes the Effect of Poly(dA:dT) on the Activation of JAK/STAT Signaling Pathway

**Figure 10:**
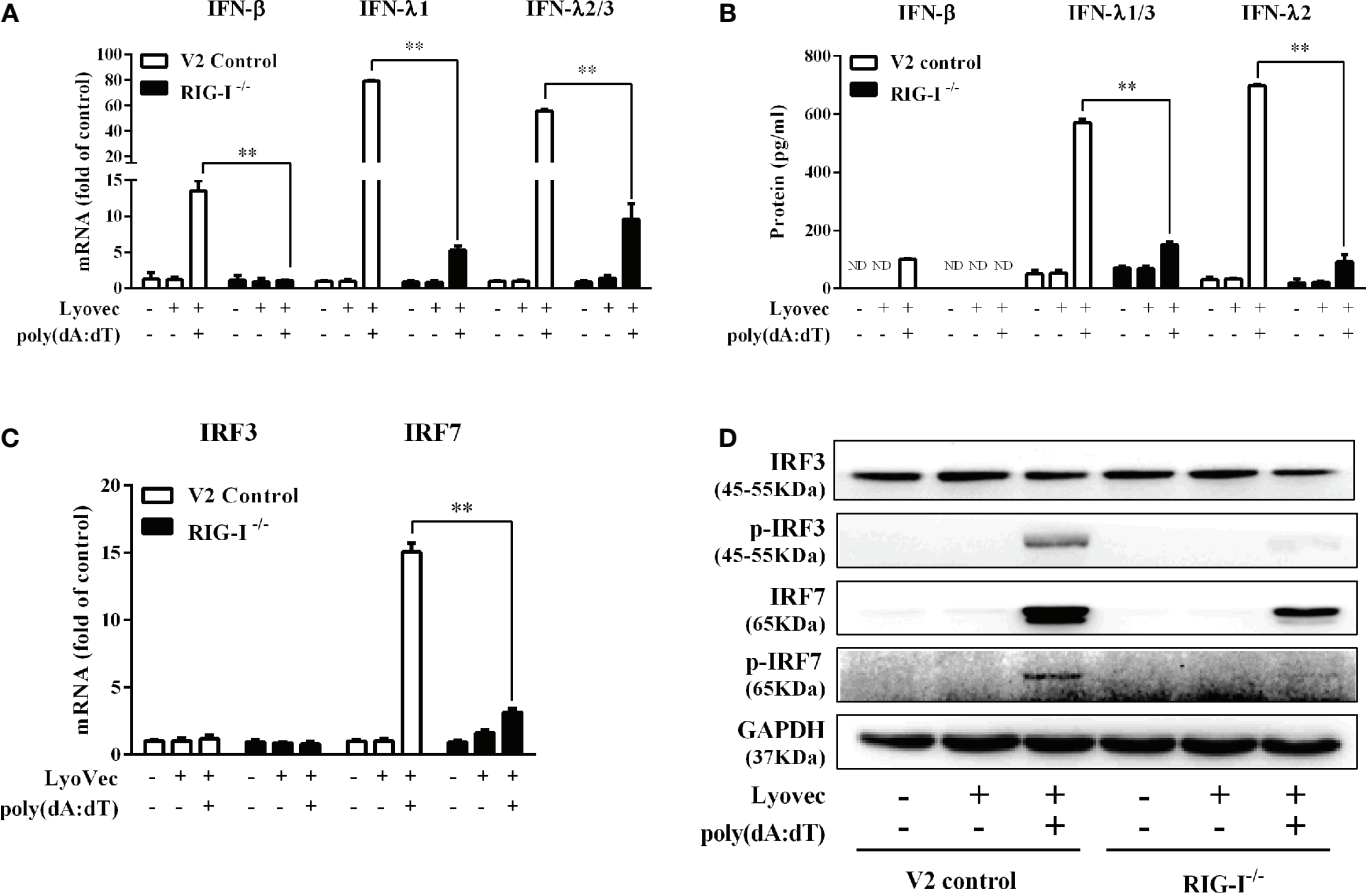
Effect of RIG-I knockout on poly(dA:dT)-induced expression of IFNs and IRFs. End1/E6E7 control cells (V2 control) and RIG-I knockout End1/E6E7 cells (RIG-I^−/−^) were transfected with or without poly(dA:dT) (0.5μg/ml) for 12h. **(A, C)** Total cellular RNAs were extracted and subjected to the real-time PCR for IRF3, IRF7, IFN-β, IFN-λ1, and IFN-λ2/3 mRNA expression. **(B)** The cell-free supernatant was collected and subjected to ELISA assay to determine IFN-β, IFN-λ1/3, and IFN-λ2 protein levels. **(D)** Total cellular proteins were collected and subjected to Western blot with antibodies against IRF3, IRF7, p-IRF3, p-IRF7, and GAPDH. The results are the mean ± SD of three independent experiments. Asterisks indicate that the differences between the indicated groups are statistically significant (ND, not detected, **P < 0.01).

**Figure 11:**
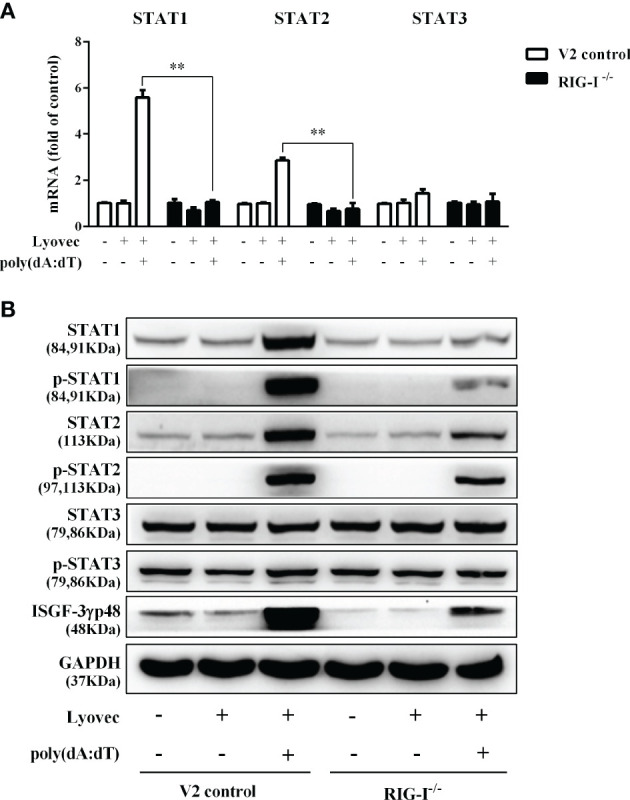
Effect of RIG-I knockout on poly(dA:dT)-induced expression of STATs. End1/E6E7 control cells (V2 control) and RIG-I knockout End1/E6E7 cells (RIG-I^−/−^) were transfected with or without poly(dA:dT) (0.5μg/ml) for 12h. **(A)** Total cellular RNAs were collected and subjected to the real-time PCR for STAT1, STAT2, and STAT3 expression. **(B)** Total cellular proteins were collected and subjected to Western blot with the antibodies against STAT1, STAT2, STAT3, p-STAT1, p-STAT2, p-STAT3, ISGF-3γp48, and GAPDH. The results are the mean ± SD of three independent experiments. Asterisks indicate that the differences between the indicated groups are statistically significant (**P < 0.01).

**Figure 12:**
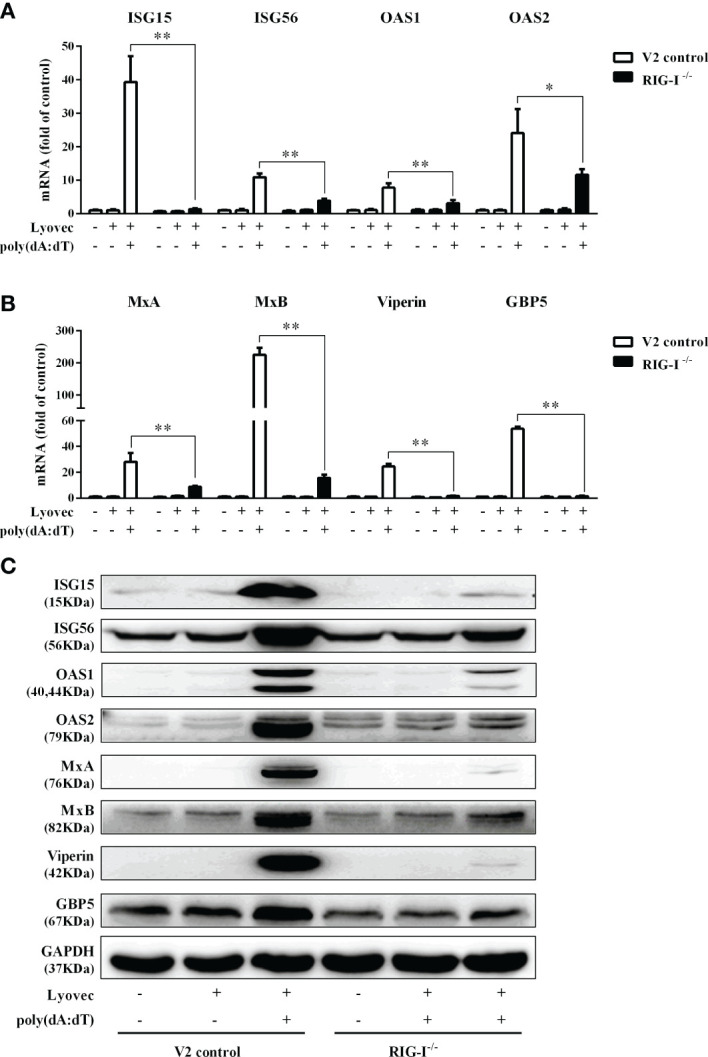
Effect of RIG-I knockout on poly(dA:dT)-induced ISGs expression. **(A, B)** End1/E6E7 control cells (V2 control) and RIG-I knockout End1/E6E7 cells (RIG-I^−/−^) were transfected with or without poly(dA:dT) (0.5μg/ml) for 12h. Total cellular RNAs were extracted and subjected to the real-time PCR for ISG15, ISG56, OAS1, OAS2, MxA, MxB, viperin, and GBP5 expression. **(C)** V2 control and RIG-I^−/−^ cells were transfected with or without poly(dA:dT) (0.5μg/ml) for 24h. Cellular proteins were collected and subjected to Western blot with the antibodies against ISG15, ISG56, OAS1, OAS2, MxA, MxB, viperin, GBP5, and GAPDH, respectively. The results are the mean ± SD of three independent experiments. Asterisks indicate that the differences between the indicated groups are statistically significant (*P < 0.05, **P < 0.01).

To determine whether RIG-I is vital for poly(dA:dT)-mediated IFN induction, we examined the levels of IFN-β and IFN-λ in RIG-I knockout cells (RIG-I^−/−^) and control cells (V2 control) transfected with poly(dA:dT). As shown in **Figures 10A, B**, IFN-β and IFN-λ levels in RIG-I^−/−^ cells were significantly lower than those in V2 control cells. In addition, we examined the impact of RIG-I knockout on the expression of IRF3 and IRF7, the key regulators of IFNs. We observed that there was a significant decrease in the levels of p-IRF3 and p-IRF7 in RIG-I^−/−^ cells as compared to those in V2 control cells (**Figure 10D**). To determine whether the suppression of IFN-β, IFN-λ, and IRFs in RIG-I^−/−^ cells is associated with the inhibition of JAK/STAT signaling pathway, we analyzed the effect of poly(dA:dT) on STATs expression. **Figure 11** demonstrated that poly(dA:dT)-mediated induction of p-STAT1, p-STAT2, and ISGF-3γp48 diminished in RIG-I^−/−^ cells as compared to V2 control cells. In addition, RIG-I^−/−^ cells had lower expression of poly(dA:dT)-stimulated ISGs (ISG15, ISG56, OAS1, OAS2, MxA, MxB, Viperin, and GBP5) than V2 control cells at both mRNA (**Figures 12A, B**) and protein (**Figure 12C**) levels.

## Discussion

As the first line of defense in FRT, human cervical epithelial cells are in direct contact with the virus and thus play an important role in preventing viral infections, including HSV-2. HSV-2 is a dsDNA virus which can be recognized by the cytosolic DNA sensors. There are several cytosolic dsDNA sensors involved in the host cells innate immunity against viral infections, including cGAS, whose secondary messenger cyclic GMP-AMP (cGAMP) can activate downstream sensor protein stimulator of IFN genes (STING). In addition to cGAS (33–35), AIM2, IFI16, and DAI are the cytosolic DNA receptors, activation of which could induce innate immune response to virus infections (36–39). Studies have shown that classical B form dsDNA is a potent immune stimulator when presents in the cytosol (40, 41). In the present study, we examined whether poly(dA:dT) has the ability to activate the intracellular innate immunity against HSV-2 infection of human cervical epithelial cells. We found that poly(dA:dT) could significantly induce the expression of IFNs/the antiviral ISGs (**Figures 4** and **6**) and inhibit HSV-2 replication (**Figure 2**). The investigation on the mechanisms for the induction of IFNs showed (**Figures 4C, D**) that poly(dA:dT) treatment of the cells enhanced the phosphorylation of IRF3 and IRF7, the key and positive regulators of IFNs during viral infections (42). IRF3 and IRF7 phosphorylation is a crucial step in activating the IFNs-mediated antiviral immunity (43). During viral infections, IRF3 is important in the early phase of inducing the transcription of IFN-α and IFN-β, which results in the IRF7 activation. Both IFN-β, IFN-λ1 gene expression is regulated by virus-activated IRF3 and IRF7, whereas IFN-λ2/3 gene expression is mainly controlled by IRF7 (44).

In addition to the positive impact on the IRFs, poly(dA:dT) could also activate the JAK/STAT signaling pathway that is vital for IFN-mediated innate immune response. We observed that poly(dA:dT) not only enhanced the expression of STAT1, STAT2, and STAT3 (**Figure 5A**) but also facilitate the phosphorylation of STAT1, STAT2, STAT3, and ISGF-3γp48 (**Figure 5B**). Several studies (45–47) have shown that comparing with STATs 1 and 2, STAT3 is an acute phase response factor with a transient activation. Therefore, it is likely that activation duration of STAT3 is shorter than that of STAT1 and STAT2. Furthermore, poly(dA:dT) treatment induced the expression of several key antiviral ISGs (**Figure 6**), including ISG15, ISG56, OAS1, OAS2, MxA, MxB, viperin, and GBP5, some of which are known to have the ability to inhibit HSV-2 infection. For example, OAS1 can directly inhibit HSV-2 proliferation (48), MxB interferes with viral replication through blocking the uncoating of viral DNA from the incoming viral capsid (49), ISG15 and ISG15-conjugates have multiple antiviral functions including inhibition of virus release and replication (50, 51). Therefore, the induction of these anti-HSV-2 ISGs provides a sound mechanism for poly(dA:dT)-mediated HSV-2 inhibition.

**Figure 13:**
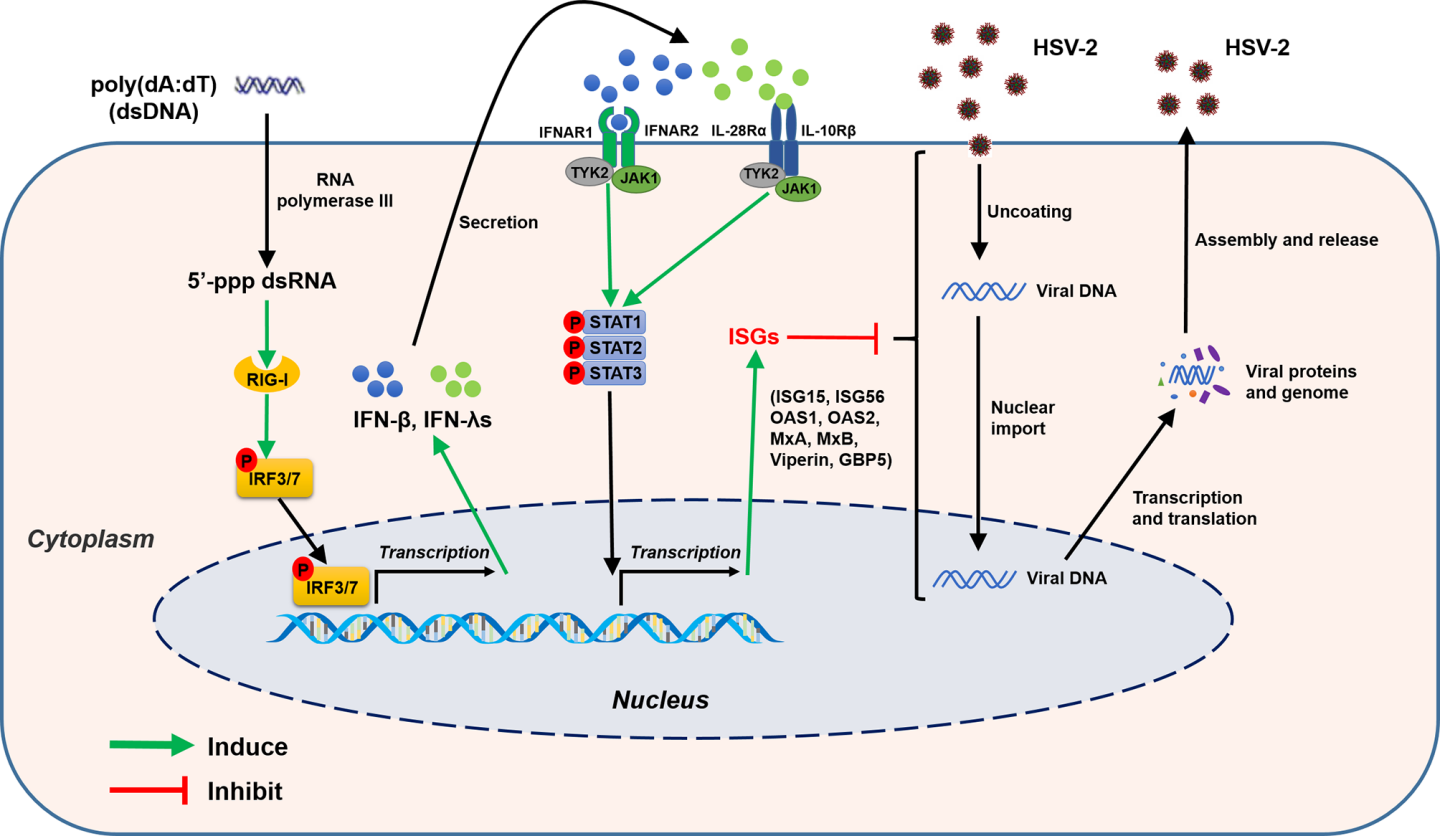
Schematic diagram of mechanisms for poly(dA:dT)-mediated HSV-2 inhibition in human cervical epithelial cells. Poly(dA:dT) can be recognized by RNA polymerase III which then converts DNA into 5’-ppp RNA, the ligand for RIG-I. The activation of RIG-I facilitates the phosphorylation and translocation of IRF3 and IRF7, resulting in the production of IFNs. The released IFNs bind to their corresponding receptors on the cell surface, and trigger JAK/STAT signaling pathway, facilitating STATs phosphorylation and inducing the antiviral ISGs (ISG15, ISG56, OAS1, OAS2, MxA, MxB, viperin, GBP5) which have the ability to block or inhibit HSV-2 at different steps of replication cycle.

To determine the initial recognition molecule(s) for the poly(dA:dT) action on HSV-2 inhibition and IFN/ISGs induction, we first examined the effect of poly(dA:dT) on the expression of the DNA sensors. We found that poly(dA:dT) had little effect on the expression of the DNA sensors at both mRNA and protein levels (**Figures 7A, B**). In addition, we failed to identify a specific DNA sensor that plays a major role in poly(dA:dT)-mediated IFN/ISG induction (data not shown). In contrast, poly(dA:dT) could significantly induce the RIG-I expression in the cells (**Figures 7C, D**). Importantly, the vital role of RIG-I in the poly(dA:dT) actions was shown in the experiments using RIG-I^−/−^ and the parental control cells (**Figure 1A**). We observed that while poly(dA:dT) could significantly inhibit HSV-2 replication in the parental control cells, it had little effect on HSV-2 infectivity in RIG-I^−/−^ cells (**Figures 8** and **9**). In addition, RIG-I^−/−^ cells lacked the effective response to poly(dA:dT) stimulation and expressed significantly lower levels of IFN-β and IFN-λ, p-IRF3 and p-IRF7 than the control cells (**Figure 10**). Similarly, the expression of p-STAT1, p-STAT2, ISGF-3γp48, and the antiviral ISGs was lower in RIG-I^−/−^ cells as compared with that in the control cells (**Figures 11** and **12**). Finally, the important role of RIG-I in the intracellular innate immunity against HSV-2 was also supported by the observation that RIG-I levels in HSV-2-infected cells were lower than those in uninfected cells, particularly in the cells infected and transfected with poly(dA:dT). The decreased expression of RIG-I in the infected cells is likely due to the negative effects of HSV-2 on the intracellular antiviral innate immunity, which is one of the strategies for the virus to escape the host cell immune response. These combined observations indicate that RIG-I is indeed a necessary and key sensor for poly(dA:dT)-mediated activation of IFN/STAT signaling pathways and HSV-2 inhibition. Therefore, it is possible that the interaction of poly(dA:dT) with RIG-I through RNA polymerase (Pol) III is crucial in initiating intracellular anti-HSV-2 innate immunity (**Figure 13**). Chiu et al. (17) reported that poly(dA-dT) serves as a template for the *de novo* synthesis of poly(A-U) RNA by DNA-dependent RNA Pol III. They showed that Pol-III is responsible for the synthesis of the RNA from poly(dA-dT), which binds directly to RIG-I. Cheng et al. also demonstrated that RIG-I is essential for B form (AT-rich) dsDNA signaling pathway as poly(dA-dT) is converted by RNA Pol III of the host cell into a 5’ triphosphate RNA intermediate, which can be recognized by RIG-I (52).

Collectively, our study for the first time has demonstrated that the activation of the RIG-I by poly(dA:dT) could effectively inhibit HSV-2 infection of human cervical epithelial cells. While the precise cellular and molecular mechanisms for poly(dA:dT)-mediated HSV-2 inhibition remain to be determined, the induction of IFNs and the multiple antiviral ISGs should be largely responsible for much of poly(dA:dT)-mediated anti-HSV-2 activity. These findings are clinically significant as they indicate that activating the intracellular innate immunity by poly(dA:dT) has potential for the prevention and treatment of HSV-2 infection. However, future *ex vivo* and *in vivo* investigations in animal models and clinical studies are necessary in order to determine whether poly(dA:dT) is effective in activating FRT innate immunity and beneficial for protecting human cervical epithelial cells from HSV-2 infection. These future studies will be crucial for the design and development of AT-rich dsDNA-based intervention strategies to control HSV-2 mucosal transmission in FRT.

## Data Availability Statement

The raw data supporting the conclusions of this article will be made available by the authors, without undue reservation.

## Author Contributions

D-DS and F-ZM performed most experiments and analyzed the data. YL, X-QX, XW, and W-HH helped with some experiments. D-DS, F-ZM, W-ZH, and WH wrote the manuscript. D-DS, F-ZM, W-ZH, and WH reviewed and revised the manuscript. All authors contributed to the article and approved the submitted version.

## Conflict of Interest

The authors declare that the research was conducted in the absence of any commercial or financial relationships that could be construed as a potential conflict of interest.
